# Bone‐Derived PDGF‐BB Accelerates Aβ Plaque Formation and Cerebrovascular Impairment

**DOI:** 10.1002/alz.095486

**Published:** 2025-01-09

**Authors:** Jiekang Wang, Kangping Song, Xu Cao, Mei Wan

**Affiliations:** ^1^ Johns Hopkins University, Baltimore, MD USA

## Abstract

**Background:**

Blood exchange experiments in heterochronic parabionts or heterochronic plasma transfer have revealed that age‐associated changes in blood composition contribute to neurogenesis and cognitive impairment in the elderly, and multiple blood‐born pro‐aging factors have been identified as causal factors to accelerate age‐dependent brain changes. We previously demonstrate that PDGF‐BB secreted by pre‐osteoclasts in bone mediates cerebrovascular impairment during aging, but the bone‐derived PDGF‐BB in Alzheimer’s disease (AD) progression is still uncertain.

**Methods:**

MCI patients and two transgenic AD mouse models, *App‐ps1* and 5XFAD, were used to evaluated PDGF‐BB level by ELISA measurement. Moreover, heterochronic parabiosis experiments were conducted using our established “*Pdgfb^cTG^* ^”^ mice, in which *Pdgfb* was overexpressed in pre‐osteoclasts, resulting the circulating PDGF‐BB concentration is elevated about 3‐fold relative to their WT littermates. Parabiosis surgery was performed to anastomose 10‐month‐old *Pdgfb^cTG^* mice with 10‐month‐old *App‐ps1* mice. As control mouse pairs, age‐matched wild type mice were anastomosed with *App‐ps1* mice. Six weeks after the parabiosis surgery, the mice were sacrificed. Serum was collected, and the brain tissue were prepared for immunofluorescence staining.

**Result:**

Comparing with MCI and healthy people, approximately 3‐fold increase of circulating PDGF‐BB. Both APP‐ps1 and 5XFAD mice displayed approximately 2‐fold increase of circulating PDGF‐BB concentration compared to their WT littermates. In parabiosis experiments, the number and area of Aβ plaques in cortex region were greatly higher in the APP‐PS1 parabionts that were paired with *Pdgfb^cTG^* mice compared to those paired with WT mice, as indicated by ThioflavinS staining and 6E10+ immunofluorescence staining. The cortex of APP‐ps1 parabionts paired with *Pdgfb^cTG^* mice showed a significant rise in GFAP+ astrocytes compared to those paired with WT mice, indicating heightened neuroinflammation. APP‐ps1 parabionts paired with *Pdgfb^cTG^* mice also showed altered brain capillaries and greater BBB leakage in brain tissue than those paired with WT mice.

**Conclusion:**

Our findings strongly suggested that increased circulating PDGF‐BB is sufficient to accelerate AD pathogenesis. Further examining the role of PDGF‐BB in exacerbating/ accelerating brain aging and AD progression may lead to the development of circulating PDGF‐BB as a new biomarker for brain degeneration.

